# Working Together for Mental Health: Evaluation of a one-day mental health course for human service providers

**DOI:** 10.1186/1471-244X-6-50

**Published:** 2006-10-31

**Authors:** Pam Grootemaat, Cathie Gillan, Gillian Holt, Wayne Forward, Narelle Heywood, Sue Willis

**Affiliations:** 1 Centre for Health Service Development, University of Wollongong, Australia; 2 Health Promotion Service, Sydney South West Area Health Service, Australia; 3 Area Mental Health, Sydney South West Area Health Service, Australia; 4 Formerly of Area Mental Health, Sydney South West Area Health Service, Australia; 5 Area Mental Health Education, Sydney South West Area Health Service, Australia

## Abstract

**Background:**

The Working Together For Mental Health course is an 8-hour course designed to demystify mental illness and mental health services. The main target group for the course is people working in human service organisations who provide services for people with mental illness.

**Methods:**

A questionnaire was administered to all participants attending the course during 2003 (n = 165). Participants completed the questionnaire before and immediately after the course, and at three month follow-up.

**Results:**

A response rate of 69% was achieved with 114 people completing the questionnaire on all three occasions. The responses showed a significant improvement in the self-assessed knowledge and confidence of participants to provide human services to people with a mental health problem or disorder, three months after the course. There was no significant improvement in participants' attitudes or beliefs about people with a mental health problem or disorder at three month follow-up; however, participants' attitudes were largely positive before entering the course.

**Conclusion:**

The Working Together For Mental Health course was successful in improving participants' confidence and knowledge around providing human services to people with a mental health illness.

## Background

People who have experienced mental illness, living in the community, have the same need for services as other people; in particular they need non-discriminatory access to disability support services when their illness results in disability [1]. The mental health literacy of staff in agencies providing human services can impact on the availability of disability support services for people with a mental illness. Improving partnerships between the community and disability support services, consumers and specialist mental health services has been identified by the Australian Second National Mental Health Plan as essential to achieving an appropriate and coordinated system of care that meets the needs of individual consumers across the life span [1].

A survey conducted in Sydney South West Area Health Service (SSWAHS) found that while staff in non-government organisations (NGOs) had regular contact with people with a mental illness, they were often not confident about providing services to this client group and requested more information about mental illness (Robinson R. unpublished report, 2003). Additionally, anecdotal evidence indicated that some mental health clients were being inappropriately referred back to mental health when trying to access disability support services. Working Together for Mental Health is a one-day course developed by the SSWAHS Area Mental Health Education team and the SSWAHS Mental Health NGO Partnerships Program to address the need for more information and provide an avenue to improve partnerships between mental health services, human service providers and consumers.

**Figure 1:**
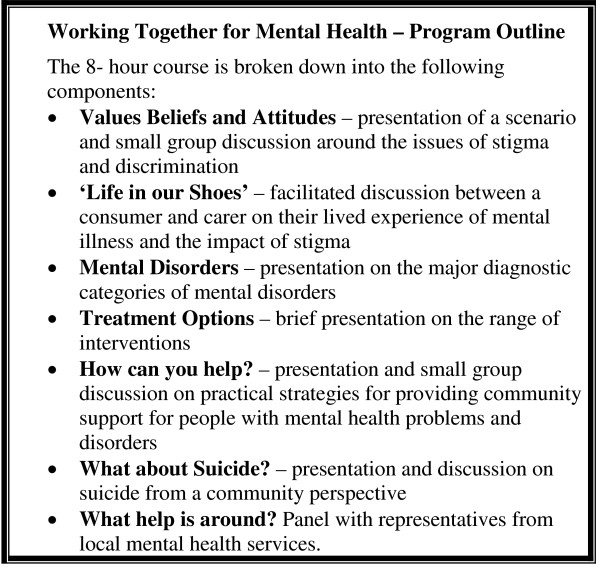
Overview of course content and teaching methods.

The Working Together for Mental Health course aims to better equip individuals in agencies providing human services to work collaboratively with consumers, families and carers, and mental health services, through having a greater personal understanding of the impact of mental illness. The course is designed to improve participants' attitudes towards people who experience mental illness as well as emphasise that participants' existing professional skills are adequate for providing human services for this client group. A key message of the course is that the support needs of people with mental illness are individual and based on abilities and disabilities, not on the presence of a mental illness *per se*. For example, assessing the housing needs of a client follows a similar process whether or not the person has a mental illness. The course also provides information on services within the South West Sydney local area and how to make appropriate referrals for clients. The primary target audience for the course is agencies that offer services used by people with a mental illness. The course is also open to other groups such as carers of people with mental illness and students. Figure 1 presents a summary of the course program and methods of delivery. More information about the course content is in the curriculum document [see Additional file 1].

An important feature of the Working Together For Mental Health course has been the involvement of mental health consumers and carers in the development, review, delivery and evaluation of the course. The course began as a generic mental health education workshop in 2001 and was revised and modified over two years in response to feedback from participants, the facilitator and consumers and carers.

The facilitator of the course has personal experience of mental illness and this has allowed information to be illustrated by drawing upon that experience. The background of the facilitator also reinforces the strengths and capabilities of consumers while challenging stigmatising attitudes. Research has shown the importance of including consumers in mental health literacy education to reduce stigma [2,3].

Since the inception of the course, written participant feedback was collected. The overwhelmingly positive nature of the feedback, along with a growing demand for the course, indicated the acceptability of the content to those attending. Additionally, mental health services were reporting improvements in their ability to work with local human service providers. In this context it was decided to undertake a pre- and post-evaluation with course participants to quantify the expected improvements.

This paper reports on the evaluation of the Working Together for Mental Health course. Specifically the evaluation aims to 1) examine whether participants' attitudes towards people with mental health problems and disorders improve after the course; 2) examine whether participants' confidence and knowledge around providing services and making referrals for consumers of mental health services improve after the course and 3) examine whether changes were maintained three months after the course.

## Methods

### Evaluation Design

A pre-, post- and follow-up self-report survey design was used to evaluate the course. A baseline questionnaire was administered on the morning of the course (pre-test) and at the end of the day (post-test). Participants were then mailed a follow-up questionnaire three months later. Questionnaires were linked using an ID number.

### Measures

The questionnaire was developed to determine the demographic profile of participants, the level of positive and negative attitudes toward people with a mental illness and knowledge and confidence to provide services to consumers of mental health services. The first section of the pre-course questionnaire requested demographic information, specifically age, sex, education level, employment status, first language and attendance at other courses about mental health. Participants were also asked about the organisation they worked for, the type of services their workplace provided and their role or position in their workplace. Participants were also asked if they had personal or family experience of a mental health problem or disorder.

The second part of the questionnaire asked a series of questions to measure positive and negative attitudes towards people with mental health problems and disorders (Table 3). A literature review identified seven survey instruments [4-10] to measure attitudes and stigma; however, none of these offered a broadly accepted measure or appeared to meet the needs of this evaluation study. A composite set of 16 questions was developed after reviewing the questions from the eight instruments and considering frequently asked questions, clarity, and the items that had previously detected a change. The questions selected were from the UK Attitudes to Mental Illness Survey [6], the Mental Health Questionnaire [7], and the New Zealand Like Minds awareness campaign survey [4]. Permission to use the questions was obtained. Two questions (13 &14) were developed by the evaluation team. Only the face validity of the resulting 16-item instrument was considered.

**Table 3:** Results of Cochran's Q and Mcnenar's Testing for questions about participants attitudes towards people who have a mental health problem or disorder.

	% of desired responses			Cochran's Q	McNemar's Test (α = 0.05)		
	**Pre**	**Post**	**Follow-up**	**Significance (α = 0.05)**	**Pre – Post p value**	**Post – Follow-up p value**	**Pre – Follow-up p value**

**Questions that the course is designed to promote agreement or strong agreement with.**							

1	Mental illness can happen to anybody						
	**99**	**97**	**99**	**0.165**	**-**	**-**	**-**
10	As far as possible, mental health services should be provided through community based facilities						
	**74**	**80**	**74**	**0.191**	**-**	**-**	**-**
13	People with schizophrenia can work in regular jobs						
	**81**	**91**	**86**	**0.003**	**0.004**	**0.1430**	**.077**
14	I could be friends with someone who has had schizophrenia						
	**94**	**97**	**97**	**0.174**	**-**	**-**	**-**

**Questions that the course is designed to promote disagreement or strong disagreement with.**							

2	People who have a mental illness are more likely than other people to be dangerous						
	**70**	**88**	**71**	**0.000**	**<0.001**	**<0.001**	**0.860**
3	There is something about people with mental illness that makes it easy to tell them from normal people						
	**82**	**85**	**84**	**0.710**	**-**	**-**	**-**
4	Once a person has a mental illness they are always unwell						
	**90**	**94**	**91**	**0.228**	**-**	**-**	**-**
5	People who have had mental illness are never going to contribute much to society						
	**97**	**98**	**97**	**0.641**	**-**	**-**	**-**
6	Someone with a mental illness is more likely than other people to have poor personal hygiene						
	**85**	**82**	**85**	**0.625**	**-**	**-**	**-**
7	If I got a mental illness I would feel I was to blame						
	**78**	**79**	**78**	**0.947**	**-**	**-**	**-**
8	It is frightening to think of people with mental illness living in residential neighbourhoods						
	**91**	**94**	**94**	**0.390**	**-**	**-**	**-**
9	I would not want to live next door to someone who has been mentally ill						
	**95**	**92**	**93**	**0.549**	**-**	**-**	**-**
11	As soon as a person shows signs of a mental illness, he/she should be hospitalised						
	**93**	**95**	**91**	**0.157**	**-**	**-**	**-**
12	I would feel uncomfortable talking to someone with a mental illness						
	**93**	**91**	**93**	**0.676**	**-**	**-**	**-**
15	I would not want to have a colleague who had schizophrenia						
	**88**	**88**	**93**	**0.102**	**-**	**-**	**-**
16	I would not want any of my children to get into a relationship with someone who had schizophrenia, even if the person had recovered						
	**66**	**67**	**66**	**0.947**	**-**	**-**	**-**

The third part of the questionnaire was a set of 13 questions developed by the study team. The questions asked about the confidence of participants in providing human services to clients with mental illness and working with mental health services. Others asked for a self-assessment of knowledge around the how, when and where to refer clients with a mental illness. The first nine questions related to the confidence and knowledge of the participants; however the final four questions focused on participants rating of the agency they worked for. Table 4 lists the confidence and knowledge questions.

**Table 4:** Results of Cochran's Q and Mcnenar's Testing for questions about participants confidence and perceived ability to work with clients who have a mental health problem or disorder.

	**% of Desired Responses**			**Cochran's Q**	**McNemar's Test (α = 0.05)**		
	**Pre**	**Post**	**Follow-up**	**Significance (α = 0.05)**	**Pre – Post p value**	**Post – Follow-up p value**	**Pre – Follow-up p value**

**Questions that the course is designed to promote agreement or strong agreement with.**							

17	I am confident that I can provide support to someone who has a mental illness						
	**81**	**92**	**92**	**0.001**	**0.009**	**1.000**	**0.001**
18	I am aware of the role GPs play in providing treatment to people with a mental illness						
	**68**	**84**	**83**	**<0.001**	**<0.001**	**0.700**	**<0.001**
19	I know how to make a referral to the local mental health service						
	**69**	**89**	**86**	**<0.001**	**<0.001**	**0.383**	**<0.001**
21	I assess the needs of a client with a mental illness the same way as I assess any other client's needs						
	**55**	**76**	**66**	**<0.001**	**<0.001**	**0.049**	**0.049**
22	I am aware of the symptoms or behaviours that would prompt me to refer a client to the mental health service or a GP						
	**65**	**91**	**86**	**<0.001**	**<0.001**	**0.307**	**<0.001**
23	I have a good understanding of services available in my work locality for people with a mental illness						
	**59**	**84**	**86**	**<0.001**	**<0.001**	**0.690**	**<0.001**
24	I am able to work in partnership with the mental health service to support a client						
	**76**	**91**	**88**	**<0.001**	**<0.001**	**0.359**	**<0.001**
25	I am able to work in partnership with a GP to support a client with a mental illness						
	**63**	**84**	**81**	**<0.001**	**<0.001**	**0.607**	**<0.001**
26	There is a good capacity in my agency to provide support services to clients who have a mental illness						
	**56**	**80**	**71**	**<0.001**	**<0.001**	**0.0120**	**.002**
28	The needs of people with a mental illness can be addressed by my agency						
	**60**	**78**	**66**	**<0.001**	**<0.001**	**0.009**	**0.216**

**Questions that the course is designed to promote disagreement or strong disagreement with.**							

20	I do not have adequate training or skills at this time to provide support to people with a mental illness						
	**35**	**59**	**61**	**<0.001**	**<0.001**	**1.000**	**<0.001**
27	People with a mental illness do not require the types of services that my agency provides						
	**83**	**79**	**89**	**0.499**	**0.499**	**0.151**	**0.152**
29	My agency is not appropriate for people living with schizophrenia						
	**62**	**72**	**75**	**0.012**	**0.029**	**0.626**	**0.007**

A Likert scale was used to capture participants' responses to the questions from parts two and three of the questionnaire. The Likert scale included the response categories: 'strongly agree', 'agree a little', 'disagree a little', 'strongly disagree' and 'don't know'.

### Recruitment

People were invited to attend the Working Together for Mental Health course by sending flyers directly to human service agencies in the local government area in which the course was to be offered. Additionally, because the course had been running for a number of years, some participants heard about it from work colleagues who had previously attended.

Questionnaires were given to all participants who attended the six courses during 2004. Participants were given a consent form that included a space to write details for posting out a follow up questionnaire. Demographic information was only collected in the pre-course questionnaire. Participants received a reminder telephone call if they had not returned the 3-month follow-up questionnaire three weeks after it was sent out.

### Ethics

This evaluation was given approval as a quality improvement project not requiring a full review by the SSWAHS Human Research Ethics Committee.

### Data Analysis

Data was entered and checked in Microsoft^® ^Access 2000 and then exported to SPSS [11] for analysis. Descriptive statistics were used to analyse the data on demographics, workplace characteristics and personal or family experience with mental health problems and disorders. To manage missing data with the attitude questions and the confidence and knowledge questions; it was assumed there was no change from the pre-test responses at post and follow-up. This allowed the inclusion of all data collected.

To test for participants' response to each question, responses were recoded to produce two categories. For questions that the course was designed to promote agreement with, the "strongly agree" and "agree a little" response was coded as one (1) with other responses coded as zero (0). For questions that the course was designed to promote disagreement with, "strongly disagree" and "disagree a little" were coded as one (1) with the remaining responses coded as zero (0). This created two categories for 'desired response' and 'other'. Cochran's Q and McNemar's tests were then used to determine the proportion of 'desired responses' for each question and how that changed between testing points. A p value of less than 0.05 was taken as denoting statistical significance.

Participants' overall attitude and perceived confidence and knowledge were also observed to see how that changed over time. Responses were recoded into a continuous, ordinal range of responses to develop a score. Participants were scored on their attitude (part two of the questionnaire) and their confidence and knowledge (part three of the questionnaire) about helping a person with a mental health problem or disorder. Those participants with the most positive attitude or self reported confidence and knowledge in response to a specific question were given a score of two (2). The next level of response was given a score of one (1) and don't know was given a score of zero. The most negative in attitude and those who felt the least confident or knowledgeable in giving help were given a score of -2 with -1 given to the next level up of response. There were 16 questions on attitude and so a total score of between 32 and -32 was possible. There were 13 questions about self reported confidence and knowledge, giving a total possible score of between 26 and -26 for these questions. The Friedman test was used to test for significant differences between the median attitude scores and median confidence and knowledge scores between testing points. A p value of less than 0.05 was taken as denoting statistical significance. Post hoc testing was conducted using the Wilcoxon test to make non-directional pair wise comparisons between each testing point. To account for increased Type 1 error the critical alpha was adjusted using Bonferroni's inequality, giving a p value of less than 0.016 as denoting statistical significance [12].

A further conservative analysis was also conducted by removing data for all participants with any missing data. This included carers and students, who were asked not to answer the third part of the questionnaire on confidence and knowledge in the workplace. This left the human service providers with complete pre-, post- and follow-up data. The above analysis was then repeated to see if there were any differences.

## Results

Over the six courses, 165 people received a set of questionnaires and a consent form. Of these 152 people (92.1%) handed back a pre-course questionnaire and a consent form indicating their willingness to participate. 145 participants (87.9%) handed back their post course questionnaire and 119 (72.1%) returned the three-month follow up questionnaire. There were a small number of participants who neglected to hand in a post course questionnaire but who did return a follow-up questionnaire. This resulted in 114 people (69%) completing the questionnaire on all three occasions.

Of the 152 participants who participated in the trial 23 (15%) participants only completed the first two sections of the questionnaire, leaving out the third section on workplace confidence and knowledge, because they were not working for a human service agency. A further 35 (23%) participants had incomplete data, not returning a post and/or follow-up questionnaire. This left 94 (62%) participants with complete data for all three components of the questionnaire.

Of those who agreed to participate, 84.9% were female, 72.4% were aged 36 years or more, 46.7% had a university or postgraduate education, 56.6% worked fulltime and 78.3% of participants spoke English as their first language (Table 1). Thirty four participants (22.4%) indicated they had personally experienced a mental illness and 73 (48%) indicated they had a family member with a mental illness. Twenty three (15%) participants were not working for a human service agency. These were primarily carers and students.

**Table 1:** Participant demographics

**Demographic**	**N**	**(%)**
Gender		
Female	129	(84.9)
Male	23	(15.1)

Age Group		
18–25 years	16	(10.5)
26–35 years	26	(17.1)
36–45 years	40	(26.3)
46–55 years	46	(30.3)
56 or more years	23	(15.1)
not stated	1	(0.7)

Education		
Postgraduate	22	(14.5)
University	49	(32.2)
TAFE	49	(32.2)
Year 11 or 12	15	(9.9)
Year 10 or below	17	(11.2)

Employment Status		
Full time	86	(56.6)
Part time	41	(27.0)
Casual	7	(4.6)
Student	6	(3.9)
Volunteer	10	(6.6)
Retired	2	(1.3)

First Language		
English	119	(78.3)
Other European	14	(9.2)
Asian Languages	15	(9.9)
Other	4	(2.6)

Support worker's made up 34% (n = 52) of participants and a further 27.6% (n = 42) of participants described themselves as Professional. Participants gave 153 different organisations where they worked, with approximately 54% being NGOs. The three main services provided by organisations were disability support services, child and family services and aged care services (Table 2).

**Table 2:** Participants' workplace characteristics

**Workplace Characteristic**	**N**	**(%)**
Type of Organisation		
Non-government organisation	82	(53.6)
Non-health government organisation	27	(17.6)
NSW health service	33	(21.6)
Other, insufficient information or not stated	11	(7.2)

Work Role		
Support Worker	52	(34.2)
Professional	42	(27.6)
Manager/Coordinator	20	(13.2)
Admin/Reception	13	(8.6)
Student	4	(2.6)
Other	14	(9.2)
Not stated	7	(4.6)

Services provided*		
Disability support	63	(18.2)
Child and family	44	(12.7)
Aged care	40	(11.5)
Employment	33	(9.5)
Counselling	32	(9.2)
Housing/Homelessness	32	(9.2)
Welfare	32	(9.2)
Youth	29	(8.4)
Legal/Justice	12	(3.5)
Other	30	(8.6)

*Most organisations provide more than one type of service.

Note: 23 people did not complete the last section of the questionnaire because they do not work for an agency that provides human services. This initially appears incompatible with only 11 people responding in the 'other' category in response to the question on the type of organisation worked for. However it is likely that carers or students identified a workplace not related to the human service field because the categories used were broad. That 25 people were in the 'student', 'other' or 'not stated' category for work role is consistent with people not working for a relevant organisation.

Participants tended to have positive attitudes to people with a mental illness (Q1–16, Table 3), with no significant difference between pre-, post- and follow-up testing. Responses to only two questions differed from this pattern; however, the positive changes were not maintained at the three-month follow-up. Question 2 asked participants' about their agreement with the statement that 'people with a mental illness are more likely than other people to be dangerous'. After the course there was a significant increase in participants disagreeing with the statement (p< 0.001) however, this was not maintained at three-month follow-up (p = 0.860). Question 13 asked participants whether they agreed or disagreed that 'people with schizophrenia can work in a regular job' and while more people agreed with the statement after the course (p = 0.004), this was not maintained at 3-month follow-up (p = 0.077).

Participant assessment of individual confidence and knowledge to support clients with a mental illness (Q17–Q25) (Table 4), showed a consistent significant improvement from pre course to the three-month follow-up. For the majority of questions there was a slight drop from the post-course values to the three month follow-up but the difference between pre and follow-up remained significant (p < 0.05). The most dramatic change was seen in response to the statement 'I do not have adequate skills and training to support a client with a mental illness' (Q20). Only 35% of participants disagreed or strongly disagreed with this statement before the course and this figure rose to 59% after the course (p < 0.001) and 61% at three-month follow-up (p < 0.001). When participants were asked whether they assessed the needs of a client with a mental illness in the same way as any other clients needs (Q21) there was a significant increase in participants agreeing with this statement after the course (p < 0.001), however agreement with this statement was only just maintained at three-month follow-up (p = 0.049).

Questions 26 to 29 (Table 4) asked participants about the ability of the agency they worked for to provide services to clients with a mental illness. While two of the four questions showed a significant improvement from pre to follow-up (p < 0.05), for the other two questions improvement was not maintained. There was a significant increase (p < 0.001) in participants who agreed that there was good capacity in their agency to provide support services to clients who have a mental illness (Q26) and this was maintained at three month follow-up (p = 0.002). The majority of participants at pre-, post- and follow-up disagreed with the statement that people with a mental illness do not require the types of services that their agency provided (Q27). After the course there was a significant increase in participants (p < 0.001) who agreed that the needs of people with a mental illness could be addressed by their agency (Q28) but this was not maintained at three month follow-up (p = 0.216). There was a significant increase in participants who disagreed with the statement 'my agency is not appropriate for people living with schizophrenia' (Q29) after the course (p = 0.029) and at three-month follow-up (p = 0.007) (Table 4).

In addition to the above testing a further analysis was conducted removing all participants who had any missing data. Also those participants who did not answer the second part of the question on confidence and knowledge in a human service provision setting were removed. Those who did not answer this section of the questionnaire were students or carers who do not work in a human service provision environment. This left a total of 94 participants. Testing did not show any real change in answers to questions about attitudes. Also Cochran Q and Mcnemar's test showed little change. The only change in significance was for Cochran's Q testing for question 13, which became insignificant (p = 0.116), however, this did not represent any change in the overall results for participants' attitudes. There were also no changes in significance levels for questions about confidence and knowledge, except for question 29. This question previously showed a just significant change between pre, post and follow up testing (p = 0.012) but changes became insignificant after removing all participants with any missing data (p = 0.081).

**Figure 2:**
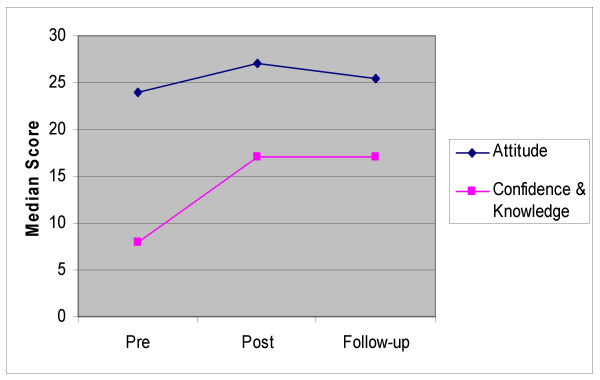
Median scores for Attitude and Confidence & Knowledge Testing. This figure is a graphical representation of the trend of median scores for pre, post and follow-up testing using the Friedman test.

Results of the Friedman tests indicated that while attitude scores improved after the course it fell back to pre-course levels at follow-up testing whereas the increase in participants' confidence and knowledge scores at post course testing was maintained at follow-up testing. There was a significant difference in the median attitude score of participants over the three time periods (p = 0.015) (Table 5), however, *post hoc *analysis using the Wilcoxon test indicated that while the post-course median attitude score (Md = 27.0) was significantly higher than the pre-course median attitude score (Md = 24.0), the follow-up median attitude score (Md = 25.5) was not significantly different to pre-course attitude indicating that improvements in participant attitude were not maintained at three months. The Friedman test also showed a significant difference in the median confidence and knowledge score over the three time periods (p < 0.001) (Table 5), however, *post hoc *testing showed that, unlike the attitude scores, both the post course median score (Md = 17.0) and the follow-up median score (Md = 17.0) were both significantly higher than the pre-course median score (Md = 8.0), indicating that improvements in self reported confidence and knowledge had been maintained at three months post-course. Figure 2 provides a graphical representation of median scores for pre-, post- and follow-up testing using the Friedman test.

**Table 5:** Results of Friedman Test for Attitude scores and Knowledge and Confidence scores.

**Participant attitudes to individuals with a mental illness.**						
					*Friedman*	
					
*Attitude Scores*	*N*	*M*	*SD*	*Md*	*χ*^2^	*p*

Pre course*	152	23.01	6.772	24.00		
Post course	152	24.70	7.330	27.00	8.351	0.015
Follow-up	152	23.98	7.042	25.50		

**Participants perceived knowledge and confidence to help someone with a mental illness.**						

					*Friedman*	
					
*Knowledge & Confidence Scores*	*N*	*M*	*SD*	*Md*	*χ*^2^	*p*

Pre course**	129	8.25	9.655	8.00		
Post course	129	15.48	8.315	17.00	77.184	<0.001
Follow-up	129	14.11	8.970	17.00		

*Significantly different from post course, p < 0.016.

**Significantly different from post course and follow-up, p < 0.016.

Further analysis was done to assess the impact of the missing data. The relationship observed for confidence and knowledge scores after using the Friedman test continued to hold up when all participants with any missing data were removed from the analysis (p < 0.001). The relationship between pre-, post- and follow-up attitude scores became only slightly significant when all cases with missing data were removed (p = 0.044). The Wilcoxon test showed that, as before, any differences for attitude were not maintained at three months, whereas these differences were maintained for confidence and knowledge testing.

The data analysis was also conducted using the approach that those with missing data were assumed to have made no change over the three testing points. Analysis of the data removing all participants with missing data showed a very similar pattern as the original analysis. Some additional analysis was conducted to assess the impact of changes in directions opposite to those expected for those participants with missing data. If these participants had responded with overwhelmingly positive responses, then results would have been amplified for confidence and knowledge results, but would also have shown that positive attitudes could have been maintained at three months. Conversely, if those participants had answered in an overwhelmingly negative way, then attitude scores would have dropped to significantly worse levels at follow-up than at pre-testing and the trend in confidence scores would have become insignificant. These results were not considered unexpected. Although an analysis cannot show every aspect of trends in the data it was felt that the main analysis described in the results gave the strongest picture of how people were responding to the questionnaire.

## Discussion

The Working Together For Mental Health course has been effective in reaching its target audience, individuals from the agencies providing human services. Participants were primarily female, older, well educated and English speaking. This participant profile is similar to that reported by Kitchener & Jorm [8] in their community trial of the Mental Health First Aid course, possibly reflecting the workplace profile from which participants were drawn. Participants were generally in professional, support worker, management or coordinating roles rather than in administrative positions. Frontline administrative positions are one of the groups considered important recipients of the course and it may be necessary to develop additional marketing strategies to reach this particular group. In addition, while there were a broad range of service providers attending the course, youth services and legal and justice services were not as well represented. Any additional marketing strategies should also aim to reach this group of service providers.

The main benefit of the course was an improvement in participants' confidence and knowledge to provide services to clients with a mental illness. A message throughout the course was that the professional skills of people in human service agencies can be applied to support people with mental illness. The improved ratings from pre to follow-up indicates that contact with clients with mental illness during 3-months of human service provision after the course, tended to maintain rather than diminish the post course confidence in their ability to provide services to this client group.

The dramatic improvement in people considering they had adequate training or skills to provide support to people with a mental illness (35% pre-course to 59% post-course and 61% at the three month follow-up) can be understood as either an artefact of the wording of the question or an indication that the course shifted the perceptions of many participants. While 81% of participants agreed or strongly agreed they are confident to provide support to people with a mental illness, only 35% indicated they had adequate skills or training to support people with a mental illness. This discrepancy could be because the training and support question was negatively worded and further testing of the questions would be needed to resolve whether this was the case. Alternatively, if the responses are an accurate indication, then although there was improvement, it remained that 39% of participants thought they needed further training and skills to support people with a mental illness. Verbal responses from participants indicated that many were interested in developing further skills and course participants have been directed towards a more comprehensive course on mental health offered by the local technical college.

It was not expected that the course would change services but rather that the perspective of the people working for the organisation could change to be more inclusive. Not surprisingly, the responses to the four questions focusing on the workplace showed less consistency in response than the questions on personal confidence and knowledge. That two questions showed changes in a positive direction from pre- to follow-up indicated that after the course, participants shifted in their perceptions of the appropriateness of mainstream service for people with mental illness. However it was disappointing that after three months the number of people that agreed that "the needs of people with a mental illness can be addressed by my agency" decreased to a level not significantly different from pre-course levels. It may be that while people agree that in theory the needs of people with mental illness can be addressed in mainstream services, the reality is not as inclusive.

A large section of the Working Together for Mental Health course focuses on attitudes and understanding what it is like to experience a mental illness or care for someone with a mental illness. Responses to the attitude questions changed little over time and the three-month follow-up showed no significant change from the pre-test responses. Initial attitudes of participants were largely positive, which may account for the lack of change but it also suggests that the course may be 'preaching to the converted' and that less time could be devoted to the issue of stigma for this target group.

Only two attitude questions showed a change from pre- to post-testing, one on dangerousness and the other on the ability of a person with schizophrenia to work in a regular job. The positive change in relation to both questions from pre- to post-indicates that attitudes in relation to ability to work and dangerousness can be shifted. However, by the three month follow-up, attitudes were not statistically different from the pre test.

The lack of a standardized tool for the assessment of attitudes towards people with mental illness means that the responses in this survey can not be compared with standardized values. Stigma remains a critical issue in relation to mental health [13] and the creation of a standardized tool for the measurement of attitudes towards people with a mental illness would be of assistance in future evaluation studies of a similar nature.

This evaluation had a number of limitations. A major limitation was that changes to work practices were not assessed, only participants ratings of their own attitudes, confidence and knowledge. It is assumed that the increase in confidence and knowledge to provide services will translate into better access to disability and other welfare and support services for people with mental illness but this was not objectively tested. Furthermore it is possible that confidence of service providers could increase but the actual quality of the service provided could diminish. A more thorough evaluation study would include some assessment of the quality of services provided to people with a mental illness though in-depth studies of actual practices.

Another major limitation of the design used is that it did not include a control group allowing for the comparison of attitudes with service providers not attending the course. Sample bias may also have affected the limited range of responses to attitude questions observed, however, small numbers and self selection of participants to attend the course made it impractical to use a random sampling technique. Further evaluation of the course may include a wait list control group.

## Conclusion

Evaluation of the Working Together For Mental Health course demonstrates that a one-day course can increase the confidence of people working outside the health sector to provide human services to people with a mental illness. The course is valuable not because it improved attitudes towards people with mental illness or taught skills, but because it raised awareness that professional skills can be used to support people with significant mental health problems.

The provision of support within the community requires that people in many different sectors and organisations can provide disability services for people who have a mental illness. This requires the building of partnerships and across agency strategies to ensure that people with mental health problems receive not only clinical services, but also the range of support services to ensure an adequate standard of living. The course was developed using principles of good practice, involving a partnership with consumers and carers during the conception, development, delivery and evaluation of the course. This evaluation has demonstrated that the product of such a strong process is able to deliver the outcomes for which it was designed.

## Competing interests

GH, WF, NH and SW were the developers of the Working Together for Mental Health course.

## Authors' contributions

PG had a major role in the design of the study, co-developed the attitudes and values questionnaire, contributed to the development of the confidence and knowledge questionnaire, administered all the questionnaires, completed the data analysis and had a major role in writing, formatting and revising the manuscript.

CG had a major role in the design of the study, co-developed the attitudes and values questionnaire, contributed to the development of the confidence and knowledge questionnaire and had a major role in writing and revising the manuscript.

GH had a role in the design of the study, contributed to the development of the attitudes and values questionnaire, co-developed the confidence and knowledge questionnaire, and had a minor role in writing the manuscript.

WF had a role in the design of the study, contributed to the development of the attitudes and values questionnaire, co-developed the confidence and knowledge questionnaire, and had a minor role in writing and revising the manuscript.

NH facilitated all the Working Together for Mental Health courses, contributed to analysis of the results and had a minor role in writing the manuscript.

SW contributed to the design of the study, facilitated the delivery of the course and had a minor role in writing and revising the manuscript.

All authors read and approved the final manuscript.

## Pre-publication history

The pre-publication history for this paper can be accessed here:



## Supplementary Material

Additional file 1 Course materials for Working Together for Mental Health. 22 page document which includes the curriculum document, references for the handouts given to participants and the PowerPoint presentation. Click here for file
