# Supplementary material for: Working Together for Mental Health: Evaluation of a one-day mental health course for human service providers
Source: BMC Psychiatry. 2006 Oct 31;6:50. doi: 10.1186/1471-244X-6-50 (PMC1635693; doi:10.1186/1471-244X-6-50)

SOUTH WESTERN SYDNEY AREA MENTAL HEALTH SERVICE

Area Mental Health Education in conjunction with Partnerships Program

Curriculum for 8 Hour Workshop

'Working Together for Mental Health'

Developed By:
- Wayne Forward (Area Mental Health Nurse Educator)
- Gillian Holt (Area Partnerships Project Officer)
- Narelle Heywood (Consumer Advocate)

In consultation with:
- Sue Willis (Area Mental Health Education Coordinator)

February 2003
Revised January 2004

Background

The need to provide basic information on mental health for community organisations and individuals has been identified by key mental health planning documents. Raising understanding and awareness about mental health is an important aspect of mental health promotion, prevention and early intervention.

The Second National Mental Health Plan (1998) identifies the need to increase mental health literacy amongst key groups in the community, including workers in government and non-government service agencies. Mental health literacy refers to information about recognition of early warning signs, how to respond to mental illness and how to get professional assistance. The Second National Health Plan also emphasises, the need for community awareness training to reduce the stigma associated with mental illness. This strategic direction is reiterated in the NSW planning document 'Caring for Mental Health: A framework for mental health care in NSW' (1998).

South Western Sydney Area Health Service has been committed to developing partnerships with community organisations in mental health through the Partnership Development Project. A number of one-day 'Understanding Mental Illness' training workshops have been held from 2000-2002 as part of this project. Around 240 people attended the workshops at 4 different venues across South Western Sydney. The workshops covered topics such as major mental health disorders, myths and stigma, consumer experiences of mental illness, treatment options and referral pathways. All workshops were fully booked. In general, the response by participants was very enthusiastic. Participants appreciated the input from mental health consumers and identified that they would have liked additional information on a number of topics. This new workshop incorporates feedback provided in the evaluations of those previous workshops.

The workshop is designed for individuals in the community interacting with people experiencing mental health problems and disorders. It is expected that it will be particularly useful for workers and volunteers from non-government and other community organisations, although it may also be helpful for staff in government organisations like the Dept of Housing and Centrelink. Input from local mental health services will be helpful in building links between those in the community and mental health service providers.

References:
Australian Health Ministers, *Second National Mental Health Plan*, Mental Health Branch, Commonwealth Department of Health and Community Services, July 1998.
NSW Department of Health Policy, *Caring for Mental Health: A Framework for Mental Health Care in NSW*, October 1998.

Workshop Overview

Target Audience
Individuals interacting with people who experience mental health problems or disorders within the wider community

Overview
A one day workshop, presented by mental health consumers and carers in collaboration with mental health professionals, for community organisations, that provides introductory information about mental health problems and disorders in an endeavour to increase the awareness of mental health issues and participants ability to work with and support people experiencing mental health problems or disorders and to work collaboratively with mental health services.

Aim
To better equip individuals in community organisations to work collaboratively with consumers, families and carers, and mental health services in providing mental health care, through having a greater personal understanding of the impact of mental illness.

Objectives
Having attended the workshop participants will be better able to:

- Identify the impact of stigma surrounding mental illness and the effect this has on mental health consumers and their families
- Identify with the experiences of someone who has, or cares for someone who, experiences mental health problems or disorders
- Understand the concept of the mental health continuum and describe the major diagnostic categorisation of mental illness
- Discuss and provide information about a variety of treatment interventions necessary to provide an holistic mental health care
- Understand the concept of collaborative partnerships in mental health care and discuss practical strategies for community organisations working with people experiencing mental health problems and their families
- Comment on issues surrounding suicide pertinent to community organisations
- Understand about the range of local clinical mental health services and local referral procedures

Resources
Resources required through the day are as follows:

- Handbook: The handbook will include SANE information sheets, information sheets developed by SWSAHS, MHS contact details
- Whiteboard and markers
- Overhead projector and screen
- LCD projector and laptop computer
- Laminated case studies and stigma questions
- Butcher's paper and pens

Program

0845 – 0900	Registration
0900 – 0920	Welcome and Introductions
0920 – 1000	Values Beliefs and Attitudes
1000 – 1030	'Life in our Shoes'
1030 – 1045	Morning tea
1045 – 1145	Mental Disorders – What are they really?
1145 – 1230	Treatment Options
1230 – 1315	Lunch
1315 – 1430	How can you help? Practical strategies for working with people with mental health problems and disorders
1430 – 1500	What about Suicide?
1500 – 1515	Afternoon Tea
1515 – 1615	What help is around? Panel with representatives from the sector health service
1615 – 1630	Summary and Evaluation

Session Aims, Objectives and Outlines

Welcome and Introduction

Overview
Presenters introduce themselves to the group and give an overview of the day's program and content. Participants have the opportunity to introduce themselves to the group.

Aim
To establish and provide participants with information regarding the workshop to assist in establishing a learning milieu

Objectives
By the end of the session participants will be given the opportunity to:
- Introduce themselves to others in the room
- State personal aims and objectives for the day
- Hear the outline of the day's program
- Develop a group learning agreement

Resources
- PowerPoint Presentation
- Program Handout

Content

Time	Content	Resources
9.00 – 9.20<br>(20 min)	• *Have PP slide displaying title of workshop as participants arrive.*<br>• Mental Health professional introduces Self and other presenters and establishes group learning agreement<br>• *Consumer or carer presenter facilitates participants introduction of themselves stating their name position, organisation and what they hope to get out of the day.*<br>• Overview of the mental health continuum and major definitions (mental illness, mental health, mental health problems, mental disorders) – see Mental Disorders session 4 aims and objectives	

Session Aims, Objectives and Outlines

Values, Beliefs and Attitudes

Overview
A presentation of a scenario and small group discussion around the issues of stigma and discrimination that affect people experiencing mental health problems and their families

Aim
Identify the impact of stigma surrounding mental illness and the effect this has on mental health consumers and their families

Objectives
At the end of the session participants will be better able to:

- Identify the negative stigma attached to mental health problems and disorders and the impact this has on mental health consumers and their families
- Identify major contributors to negative attitudes and stigma about mental illness
- Identify what can be done decrease the stigma that surrounds mental illness
- Identify the impact of culture on stigma

Resources

- PowerPoint slide
- Butchers paper and pens
- Laminated stigma questions

Content

Time	Content	Resources
9.20 – 10.00<br>40 min	Presentation of scenario of Bart<br>Small group discussion of stigma questions<br>Small groups report back to the whole group	

Session Aims, Objectives and Outlines

'Life in our Shoes'

Session Overview
A facilitated discussion between the interviewer and interviewees (consumer and carer) regarding their experiences with mental illness and the impact of stigma

Aim
Identify with the experiences of someone who has, or cares for someone, who experiences mental health problems or disorders

Objectives
By the end of the session participants will be better able to:
- Empathise with what it is like to experience a mental illness or disorder or care for someone who has.
- Describe some of the issues faced by an individual following a diagnosis of a mental illness.
- Describe some of the positive ways to support people with mental health problems and their families
- Describe the impact of having a friend or relative diagnosed with a mental illness
- Describe the issues faced by carers when caring for a friend or relative with a mental illness.

Resources Required
- Interview Questions

Session Aims, Objectives and Outlines

Session Outline

Time	Content	Resources
10.00 – 10.30<br>(25 min)	• Introduce consumer and carer to the group (name only)<br>• Outline the interview process and the appropriateness of asking questions by the group<br>• Conduct Interview following as a guide the following questions:<br><br>1. Could you tell us a little bit about yourself? (What you do with yourself and Why you have come here to day)<br><br>2. Consumer Only) Briefly could you outline what it has been like for you to experience the loss of your mental health?<br><br>3. Consumer – From your experience what can you tell us about the way that people who have a mental health problem are treated? Carer – How difficult was it for you as a carer to support your family member?<br><br>4. Are you aware of ever having experienced stigma or discrimination because of mental health issues?<br><br>5. What positive things can people do to support you?<br><br>6. What is one thing you would like people to know about mental illness?	

Session Aims, Objectives and Outlines

Mental Disorders – What are they really?

Overview
Following the brief introduction given in the welcome about the mental health continuum, mental health as a concept is explored and a brief summary given of the major diagnostic categories used in mental health care

Aim
Understand the concept of the mental health continuum and describe the major diagnostic categorisation of mental illness

Objectives
At the end of this session participants will be better able to:
- Understand the concept of the mental health continuum and define mental health
- Describe what is meant by the following terms
  - Schizophrenia
  - Depression
  - Bi-Polar Affective Disorder
  - Anxiety Disorders
  - Personality Disorders
- Describe the impact of different symptoms on an individual's life

Resources
- PowerPoint Presentation
- Handouts

Content

Time	Content	Resources
1045 - 1145<br>60 min	• Go through PowerPoint Presentation, using stories and personal examples to illustrate symptoms and their impact<br>• Period of time allowed for questions following the presentation of each diagnostic category	PowerPoint Presentation and Handouts

Session Aims, Objectives and Outlines

Treatment Options

Overview
A presentation of the variety of interventions that can be used in the treatment of mental health problems or disorders necessary to provide a holistic mental health care

Aim
Discuss and provide information about a variety of treatment interventions necessary to provide holistic mental health care

Objectives
By the end of the session participants will be better able to:
- Describe the role, range and impact of medical based interventions
- Describe the role of psychological and psychosocial interventions
- Explore the importance of psychosocial rehabilitation
- Understand the importance of consumer and carer empowerment and the role of advocacy

Resources
- PowerPoint Presentation

Outline

Time	Content	Resources
1145 – 1230<br>45 mins	• Work through the PP Presentation<br>• Period of time allowed for questions following the presentation of each slide	

Session Aims, Objectives and Outlines

How can you help?

Overview
A brief presentation on the role of partnerships and skills for working with people experiencing mental health problems and disorders. Following this small groups work on individual scenarios allowing participants the opportunity to discuss and develop some practical strategies that they can use in their workplace to assist them to better work with people experiencing a mental illness or disorder

Aim
Understand the concept of collaborative partnerships in mental health care and discuss practical strategies for community organisations working with people experiencing mental health problems and their families

Objectives
At the completion of the session participants will be better able to:
- Describe the role and range of government and non-government organisations working in partnership to provide services to people experiencing mental health problems and their families
- Describe positive ways to communicate and interact with people with mental health problems and disorders
- Describe positive ways in which a community organisation can assist a person with a mental health problem or disorder

Resources
- PowerPoint Presentation
- Laminated Scenarios and Questions
- Butchers paper and pens

Outline

Time	Content	Resources
1330 – 1430<br>60 min	• Work through the PP Presentation<br>• Period of time allowed for questions following the presentation of each slide<br>• Work through scenarios in small groups and summarise small group discussion back to the large group at conclusion	

Session Aims, Objectives and Outlines

What about Suicide?

Overview
A brief look at the issue of suicide from a community perspective

Aim
Comment on issues surrounding suicide pertinent to community organisations

Objectives
At the end of the session participants will be better able to:
- List some current statistics related to suicidal behaviour, suicide attempts and deaths by suicide
- Discuss risk factors associated with suicide
- Discuss the importance of protective factors

Resources
- PowerPoint presentation
- Scenario

Outline

Time	Content	Resources
1430 – 1500<br>30 min	• Work through PowerPoint presentation<br>• Facilitate large group discussion of scenario - Fred	

Session Aims, Objectives and Outlines

What help is around?

Overview
A presentation by the local Mental Health service, outlining their role, services and facilities and the referral process to the MH service concluding with an opportunity for participants to ask questions.

Aim
Understand about the range of local clinical mental health services and local referral procedures

Objectives
At the end of the session participants will be better able to:
- Name some members of the local mental health service
- List the variety of services offered by the local mental health service
- List the phone numbers of the local Mental Health Service and referral processes

Resources
- Sector based PP presentation

Outline

Time	Content	Resources
1515 – 1615<br>60 min	• Presentation of an overview of the local Mental Health service, including philosophy of care and basic structure, and a description of each of the facilities<br>• Explanation of the referral/intake system<br>• A panel discussion allowing participants to ask questions	

Session Aims, Objectives and Outlines

Summary and Evaluation

Overview
A summary of the day will be given and an opportunity for participants to evaluate the day.

Aim
To reinforce the content and evaluate the workshop

Objectives
By the end of the session participants will be better able to
- Summarise the content of the workshop
- Reflect on the usefulness of the workshop to their professional skills and clinical practice.

Resources
- PowerPoint presentation
- Evaluation form
- Certificates of attendance

Outline

Time	Content	Resources
1615 – 1630 (15min)	• Go through PowerPoint presentation summary<br>• Pass out evaluations<br>• Pass out certificates of attendance	

Participant Handouts

Every participant receives the following

Information on mental illness
Copies of each of the SANE handouts titled:

- Mental Illness the facts
- Schizophrenia
- Bipolar Disorder
- Depression
- Treatments for Mental illnesses
- Anxiety Disorders

These can all be accessed from the web site www.sane.com.au

Stigma fact sheets

- Stigma: a facts sheet
- Rights of people living with mental health problems

The fact sheets used can be accessed from www.mentalhealth.asn.au

Web addresses
Participants are given a page providing the following web page addresses for further information.

www.health.nsw.gov.au
www.health.gov.au/hsdd/mentalhe
www.mhcs.health.nsw.gov.au
www.tmhc.nsw.gov.au
www.amhcn.com.au
www.reachout.com.au
www.beyondblue.org.au

Local service information
The local service representative that attends the course provides a local service pamphlet and information with details on how to contact there local services eg. phone numbers etc.

Mental Health

" Working together for mental health"

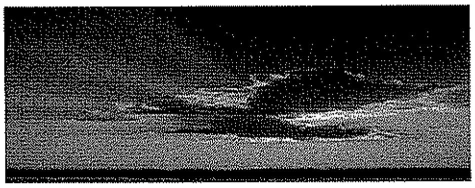

Presented By

South Western Sydney Area Mental Health Service

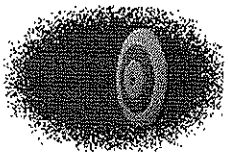

Aim

To better equip individuals in the wider community with an understanding of mental health in order to improve services, attitudes, values and beliefs held towards people who experience mental health problems or disorders.

Seminar Overview

- 'Life in My Shoes'
  10.30 – 10.45 MORNING TEA
- Mental Disorders – What are they really?
- Treatment options
  12.30 – 1.15 LUNCH
- How can you help?
- What about Suicide
  3.00 – 3.15 AFTERNOON TEA
- What help is around

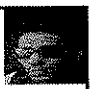

Bart is a 30 year old male who has a mental illness. He currently lives by himself in a unit block. He approaches a community service agency complaining that the neighbours have been harassing him by playing music loudly, taunting him and stealing his mail. He asks for help to deal with the neighbours.

Questions to consider

- What is the effect of stigma on people with mental disorders and their families?
- What factors contribute to stigma?
- What is the impact of cultural issues and beliefs on stigma?
- What can we do to reduce stigma?

What is the effect of stigma on people with mental disorders and their families?

What factors contribute to stigma?

What is the impact of cultural issues and beliefs on stigma?

What can we do to reduce stigma?

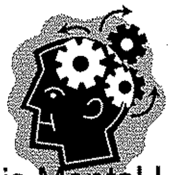

What is Mental Health?

Increased connectedness and supportiveness of communities

Emotional and social well-being

Mental Disorders
- What are they Really?

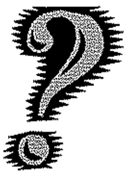

Major Disorders

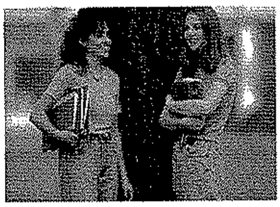

- Depression
- Anxiety
- Schizophrenia
- Bi-Polar Disorder (Manic Depression)
- Personality Disorders

Depression

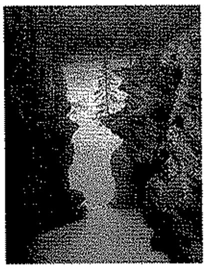

- Depression vs 'The Blues'
- Affects 1 in 5 people
- Signs & Symptoms:
  feeling sad, loss of interest, feeling worthless, sleep disturbances, changes in appetite

Anxiety

- Affects 1 in 10 people
- Includes Panic Disorder, Generalised Anxiety and PTSD

•Signs & Symptoms

•Physical – eg pounding heart, hyperventilation

•Psychological – eg worry, panic

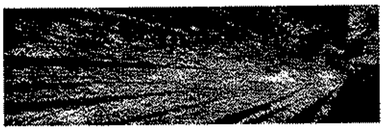

Schizophrenia

- NOT A SPLIT PERSONALITY
- Affects 1 in every 100 people
- Signs & Symptoms
  Positive: Confused thinking, delusions and hallucinations
  Negative: Poor motivation, poor self esteem, isolation

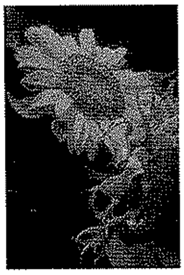

Bi-Polar Disorder

(Manic Depression)

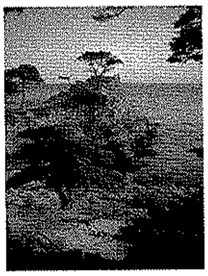

- Signs & Symptoms
  Episodes of severe depression and hypo/mania
- Affects 2 in every 100

Personality Disorders

- Very Complex group of disorders most common is Borderline Personality Disorder
- Signs and Symptoms
  Difficulty relating to others, mood swings, Impulsive behaviour, suicidal ideation/ self-harm, Often coexisting depression, anxiety or PTSD
- Affects 2 in every 100 people

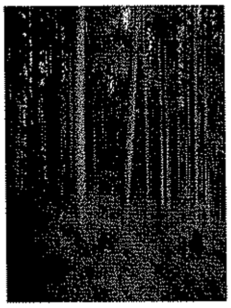

Treatment Options

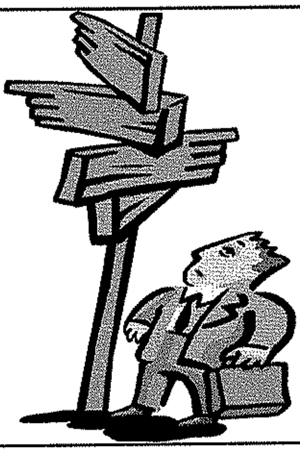

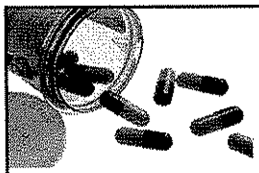

Medical Treatments

- Medications
  Anti-depressants, anti-psychotics, mood stabilisers,
  NB: Significant side-effects can be experienced such as:
  - drowsiness, muscle stiffness, weight gain, restlessness, dry mouth, sexual dysfunction
- Electro Convulsive Therapy (ECT)

Psychosocial Interventions

- Case Management
- Psychotherapy
- Family / carer support
- Disability support
- Information and education

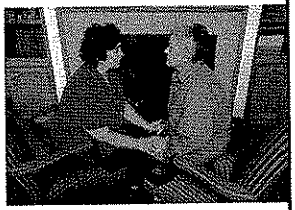

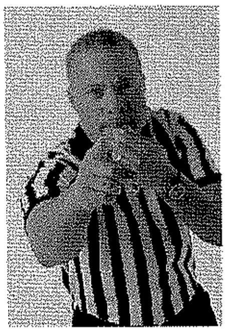

Participation and Advocacy

- Consumer Network
- Carer Network
- Community Consultative Committees (CCC)

How Can You Help?

What questions do you have about working with people experiencing a mental health problem or disorder?

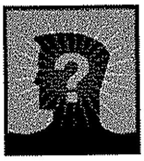

Mental Health Partnerships

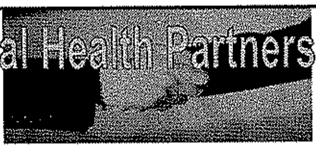

- Mental health clinical services
- Mainstream community services
- Mental health support (non-clinical) services
  - Disability support services
  - Services for family and carers

Supporting people with mental health problems

- Always treat the person with respect and dignity and in the same way you would treat anyone else
- Respect privacy and confidentiality
- Be non-judgemental
- Accept the person's reality
- Avoid confrontation

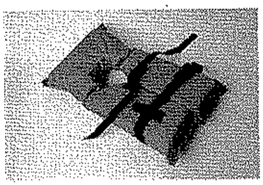

How can you help?

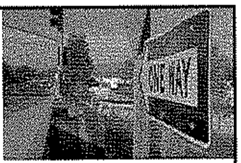

- Encourage or assist the person to seek professional advice
- Encourage activities that promote good mental health
  - Physical activity
  - Stress management
  - Development of positive coping strategies
  - Social activities
- Provide other relevant services as required

---

1. **What else would you need to know to assess and respond to this situation?**
2. **Should the client be referred to a mental health service for possible clinical intervention? If so, why?**
3. **What other factors might be impacting on this client's mental health?**
4. **What other support agencies could be involved and what role might they play?**

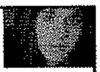

Lola, aged 45, has made contact with the local community neighbourhood centre. She is very distressed about her mother who has been becoming more difficult over the past six months. Her mother has had a mental illness for many years. Lola lives close to her mother but works and has 4 children.

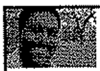

John is a 32 year old male who has schizophrenia. He approaches an employment service for assistance to get a job. John lives alone in community housing and is quite lonely. He is stable on medication. He has been unemployed since he became ill, but was a carpenter prior to his illness.

Tobias is a 17 year old male who has been admitted to the local youth refuge. He was asked to leave home because of his drug use. He has just dropped out of school. Over the past year his teachers noticed that he seemed to be quite depressed and his friend told the school counsellor that he had been talking about killing himself.

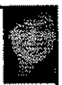

Selina is a 33 year old female who has 2 children who both go to school. She has come into a St Vincent de Paul shop to ask for some assistance because her electricity has just been cut off. Selina tells the worker that she is not sleeping well and is feeling very agitated.

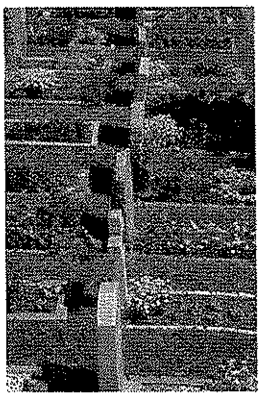

What about Suicide?

Facts and Figures

In any given year in NSW :

- up to 90,000 people may show possible suicidal behaviour
- 30,000 of these people will attempt suicide
- 700 people will die as a result

(ABS 1996)

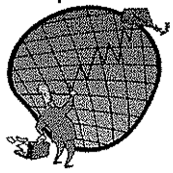

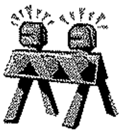

At Risk Groups

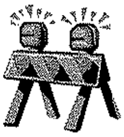

- Young and older people, males, minority groups
- Alcohol and substance abuse, mental health problem or disorder, chronic pain
- History of suicide attempts, feelings of hopelessness, major loss or lifestyle change
- Isolation, unemployed

Protective Strategies

- Strong social supports
- Optimism
- Ability to access help
- Effective coping strategies
- Problem solving ability
- Willingness to accept help

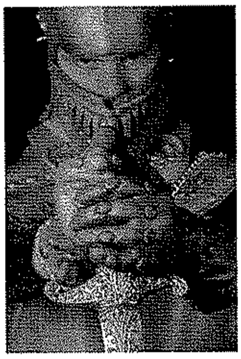

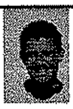

Fred is 23 years old married with one child and recently lost his job and driver's licence. He enters the neighbourhood centre to ask if he could have some money as he needed to buy some pain relief for his chronic back injury. As you are discussing with Fred the issue of the money he tells you that he wants to kill himself because life is just to much at the moment.

Further Training is Available

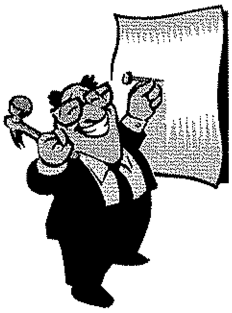

For Details Contact:

Your local

Area Suicide Prevention Coordinator

Or MH Service

More Information

Mental Health Information Service
- PH: 9816 5688 or www.mentalhealth.asn.au.

•**SANE Australia**
- 1800 688 382 or www.sane.org

• **www.mentalhelp.net**

•**www. healingwell.com**

Summary

- Values, Attitudes and Beliefs
- 'Life in my Shoes'
  - Mental Disorders
- Treatment Options
  - What about Suicide?
- How can you help?
  - What help is around?

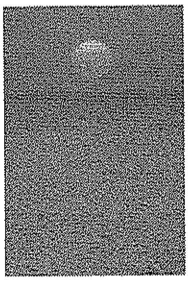

Supplement: Additional file 1 — Course materials for Working Together for Mental Health. 22 page document which includes the curriculum document, references for the handouts given to participants and the PowerPoint presentation. [file 1471-244X-6-50-S1.pdf]
